# Refractive results with SMILE using lower energy settings in the United States

**DOI:** 10.1371/journal.pone.0258835

**Published:** 2021-10-22

**Authors:** Erica T. Liu, Ruti Sella, Phillip Goernert, Kevin Kim, Henry Chen, Robert T. Lin

**Affiliations:** 1 IQ Laser Vision, City of Industry, California, United States of America; 2 Department of Ophthalmology, Rabin Medical Center, Petach Tikva, Israel; 3 Sackler Faculty of Medicine, Tel Aviv University, Tel Aviv, Israel; 4 Department of Psychology, Brandon University, Manitoba, Canada; 5 College of Osteopathic Medicine of the Pacific, Western University, Pomona, California, United States of America; Singapore Eye Research Institute, SINGAPORE

## Abstract

**Purpose:**

To report the visual and refractive results of small incision lenticule extraction (SMILE) with low energy settings in the United States (US) and to evaluate outcomes for low astigmatism treatment.

**Setting:**

Private clinical practice.

**Design:**

Retrospective cohort study.

**Methods:**

This study retrospectively reviewed 462 consecutive eyes that underwent SMILE with lower energy settings. Inclusion criteria included all patients between the ages of 19–39 with myopic astigmatism up to -11.25 diopters (D) spherical equivalent (sphere up to -10.00 D, astigmatism up to -3.00 D), and corrected distance visual acuity of at least 20/25. Eyes with low astigmatism (0.25 D-0.50 D) were also included. Outcome analysis was performed according to the Standard Graphs for Reporting Refractive Surgery at postoperative month (POM) 1, and POM 3–6 when data were available.

**Results:**

The mean preoperative spherical equivalent treated was -4.96 ± 2.07; at POM 1, 92% of eyes achieved uncorrected visual acuity (UCVA) of 20/20 or better and maintained visual stability throughout the remainder of the study. At last visit, 431 eyes (93%) achieved UCVA of 20/20 or better, and 461 eyes (99.8%) were 20/25 or better. Ninety-seven (21%) eyes gained at least 1 Snellen line of corrected distance visual acuity and no eyes lost 2 or more lines. Almost all eyes (n = 453, 98%) were within 0.5D of target; 85% of eyes with low astigmatism had ≤0.25 D at last visit compared to 80% of eyes with moderate astigmatism.

**Conclusions:**

SMILE with U.S.-approved low energy settings is safe, predictable, and efficacious and provides patients with a fast visual recovery.

## Introduction

Laser vision correction has continually evolved to include small incision lenticule extraction (SMILE), first introduced by Sekundo et al. [1]. This technique now incorporates even smaller incisions and different energy settings to increase accuracy and healing times. Incremental improvements have resulted in a greater adoption by refractive surgeons of the procedure and increased overall patient satisfaction [2]. SMILE is a flapless procedure that may have advantages for specific patient groups when compared to the gold standard refractive surgical procedure, laser in situ keratomileusis (LASIK) [3, 4]. In the United States, refractive surgeons have lagged behind their international colleagues in adapting this technology; this may have been a result of being unable to address astigmatism in early iterations coupled with a comparatively slower recovery time [5]. This may also explain the minimal amount of data in the published literature on results with SMILE using the most updated parameters available in the U.S. [6, 7].

To the authors’ knowledge, this is the first report on a large cohort of patients treated with SMILE that evaluates both refractive outcomes in patients with lower levels of astigmatism and lower energy settings on the device. The U.S. Food and Drug Administration initial recommendations for the SMILE device limited its use to the treatment of myopia only, without any recommendation about astigmatic correction [8]. Laser spot spacing (3.0 μm) and incision size (90 degrees) also were limited in the U.S. when compared to approved parameters outside the U.S. Subsequent approvals in the U.S. allowed for lower energy settings by increasing spot spacing to 4.5 μm, decreasing incision size to 60 degrees and expanded the ability to treat myopia between -1.00 to -10.00 D and astigmatism from 0.75–3.00 D [9]. The decrease in spot spacing significantly decreased total energy input into the cornea which has been shown to decrease visual recovery time [10–12].

The primary endpoint of this study was to evaluate the refractive results achieved with SMILE using lower energy settings available in the U.S. These results were compared to pivotal study outcomes in the U.S. with LASIK and SMILE [5]. Secondary outcomes were to evaluate refractive outcomes with SMILE in patients with 0.25 D to 0.50 D of astigmatism.

## Methods

This retrospective study reviewed results of patients who were treated by one of two experienced surgeons (ETL and RTL) at one surgical center (IQ Laser Vision; CA, USA) during 2019. This study was submitted to an independent Institutional Review Board (IntegReview; Austin, TX, USA) and determined to be exempt from ethical approval based on its retrospective and de-identified nature. Inclusion criteria included all patients between the ages of 19–39 with myopic astigmatism up to -11.25 diopters (D) spherical equivalent (sphere up to -10.00 D, astigmatism up to -3.00 D), and corrected distance visual acuity of at least 20/25 who had successfully undergone the SMILE procedure for myopia or myopic astigmatism. Patients’ records were excluded from inclusion if they had: thin corneas (<500 μm), proven or suspicious cases of corneal ectasia by corneal tomography, previous ocular surgeries, ocular comorbidities or scars that were likely to affect visual outcomes, pregnancy, and/or use of systemic steroids, immunosuppressants, or antidepressants.

### Preoperative evaluation

Preoperative evaluation was performed on all patients by experienced optometrists and consisted of medical and ocular history, uncorrected distance visual acuity (UDVA), best corrected distance visual acuity (CDVA), cycloplegic CDVA, slit lamp biomicroscopy, and dilated fundus exam. Patients who wanted SMILE but had astigmatism too low to program into the device were managed according to the following: astigmatic errors of 0.25 D were treated with the spherical equivalent. Astigmatic errors of 0.50 D were treated with the trial frame subjective preference of either spherical equivalence of spherical treatment only versus increasing the astigmatism treatment to 0.75 D and decreasing the sphere by 0.125 D to maintain manifest refraction spherical equivalence. For this paper, we define ‘low astigmatism’ as 0.25 to 0.5 D of astigmatism that cannot be programmed in the U.S. Topography (MM-1 Magellan; Nidek, Gamagori, Japan) and tomography (Galilei G2; Ziemer Ophthalmic Systems, Port, Switzerland) were also performed. Patients were all informed of risks, benefits, alternatives and limitations prior to proceeding with the procedure.

### Surgical procedure

The surgical procedure was performed by one of two experienced surgeons (RTL or ETL) on a 500-kHz femtosecond laser (VisuMax; Carl Zeiss Meditec, Jena, Germany). Patients were given oral diazepam 1-2mg as needed.

Patients with >2 D of astigmatism were marked at the slit lamp or with the assistance of Torsion Error Detection (TED) of the excimer laser (EC-5000; Nidek, Gamagori, Japan). Patients with ≤2 D of astigmatism were adjusted for body and head alignment to minimize impact of cyclotorsion per manufacturer recommendation. Laser settings were set at: laser cut energy index of 25–27 (125-135nJ), spot spacing of 4.5 μm (low energy), lenticule diameter of 6.5mm, cap thickness of 120 μm, and 60° superior incision. A small size treatment pack was used for all eyes. A site-specific nomogram modified from the manufacturer was used to determine laser input values from manifest refraction. When the offset resulted in a recommended treatment higher than FDA-approved parameters, the laser input values were converted to vertex of zero.

All patients were assisted onto the procedure bed with special attention paid to alignment. Initial topical anesthesia was obtained with proparacaine 0.5%. The second eye was taped close to prevent desiccation. The first eye was held open with a bladed speculum and the patient was instructed to fixate on the blinking green light until suction was applied. To assist in proper centration with the visual axis, we confirmed our alignment with the Purkinje measurement from the HD Analyzer (Visiometrics; Keeler, Malvern, PA, USA). Any discrepancy resulted in a repeat dock until alignment was consistent. Treatment pack was rotated to align axis marks to screen markings in patients with astigmatism >2 D. The lenticule creation with our settings took 23 seconds. Additional topical anesthesia was provided with 1% lidocaine after lenticule creation. The eye was fixated with forceps during the second blunt dissection portion of the procedure. Either a spoon spatula or lenticule spatula was used to release the lenticule which was then massaged out or removed with microforceps. Select eyes had pockets flushed with BSS or 0.1% dexamethasone. All caps were smoothed with a surgical eye spear sponge. At the end of the procedure, all eyes received one drop of prednisolone 1% / gatifloxacin 0.5% (Imprimis; Ledgewood, NJ, USA).

### Postoperative care

All patients were sent home with sunglasses and instructed to use preservative-free artificial tears (Oasis Tears; Oasis, San Dimas, CA, USA) every hour while awake and prednisolone 1% / gatifloxacin 0.5% QID for 1 week.

Follow up exams consisting of UDVA, slit lamp biomicroscopy, fluorescein staining, and auto-refraction were performed at postoperative day 1, week 1, month 1 (POM 1), and 3 to 6 months (POM 3–6). Manifest refraction and CDVA was performed at POM1 and POM 3–6 by the same group of experienced optometrists who performed the preoperative evaluations. Patients who did not follow up after POM 1 are defined as “lost to follow up” and patients who did follow up at POM 1 and POM 3–6 are defined as “completing follow up.” “Last visit” was defined as their visit at POM 1, if patients were lost to follow up or returned to co-managing eye care specialists, or POM 3–6 if patients completed follow up. “Final visit” was defined as patient follow-up visit at POM 3–6.

### Statistical analysis

All data were collected and entered into Datagraph-Med 5.70 software (Datagraph-med; Wendelstein, Germany) for analysis. We performed all statistical analysis using IBM SPSS Statistics version 25 (IBM; Armonk, NY, USA). Univariate logistic regression models using generalized estimating equations to adjust for inter-eye dependency were used to assess the clinical outcomes of refractive cylinder levels, spherical equivalent levels, and final UCVA. We considered a P-value of < 0.05 statistically significant. Standard Graphs for Reporting Refractive Surgery (London Vision Clinic; London, England) [13] were created with Microsoft Excel 365 (Microsoft Corp.; Redmond, WA, USA).

## Results

**Fig 1:**
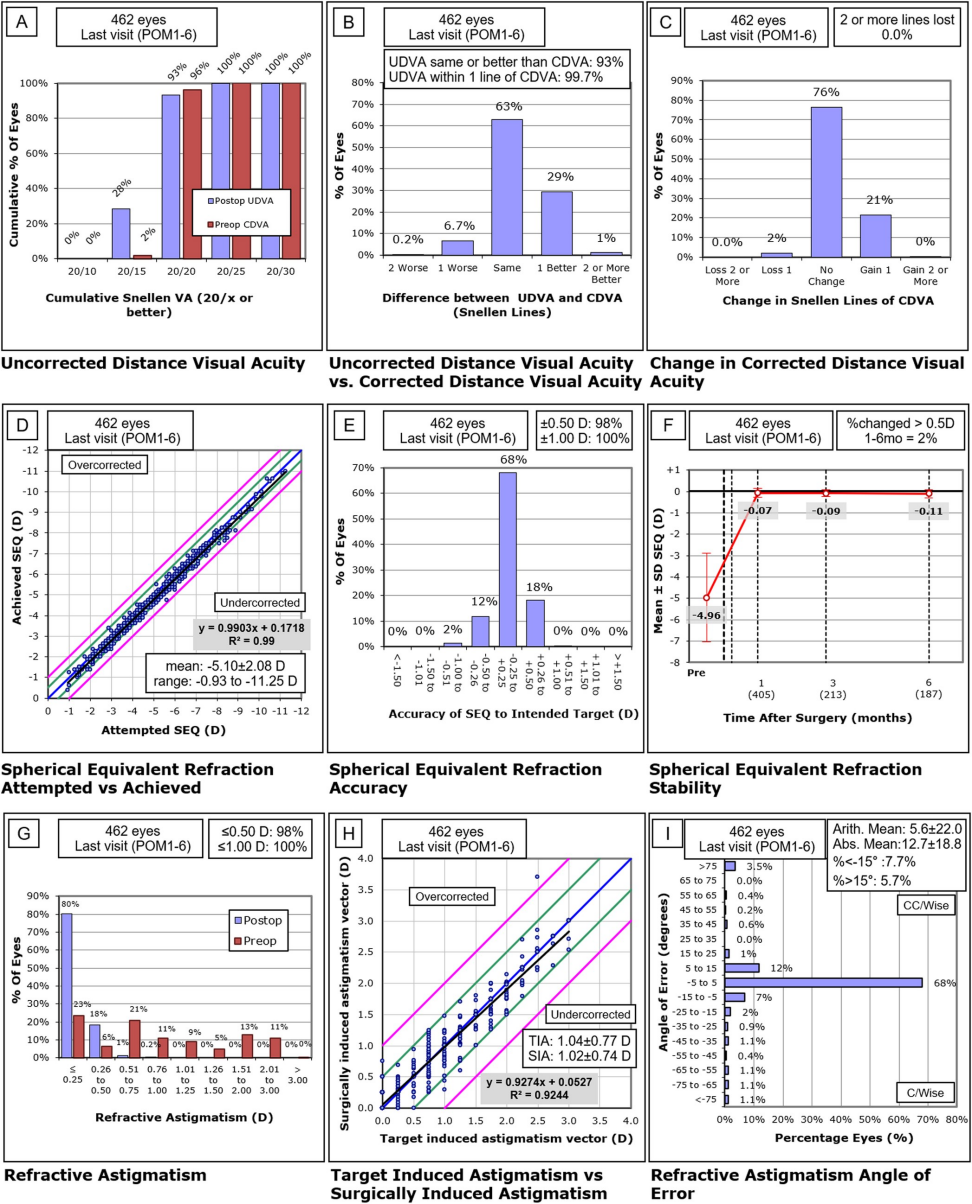
Refractive outcomes of 462 eyes at their last visit at either postoperative month 1 or 3–6 (POM 1–6) after treatment with small incision lenticule extraction. CDVA = corrected distance visual acuity; D = diopters; Postop = postoperative; Preop = preoperative; SEQ = spherical equivalent refraction; TIA = target induced astigmatism; UDVA = uncorrected distance visual acuity.

**Fig 2:**
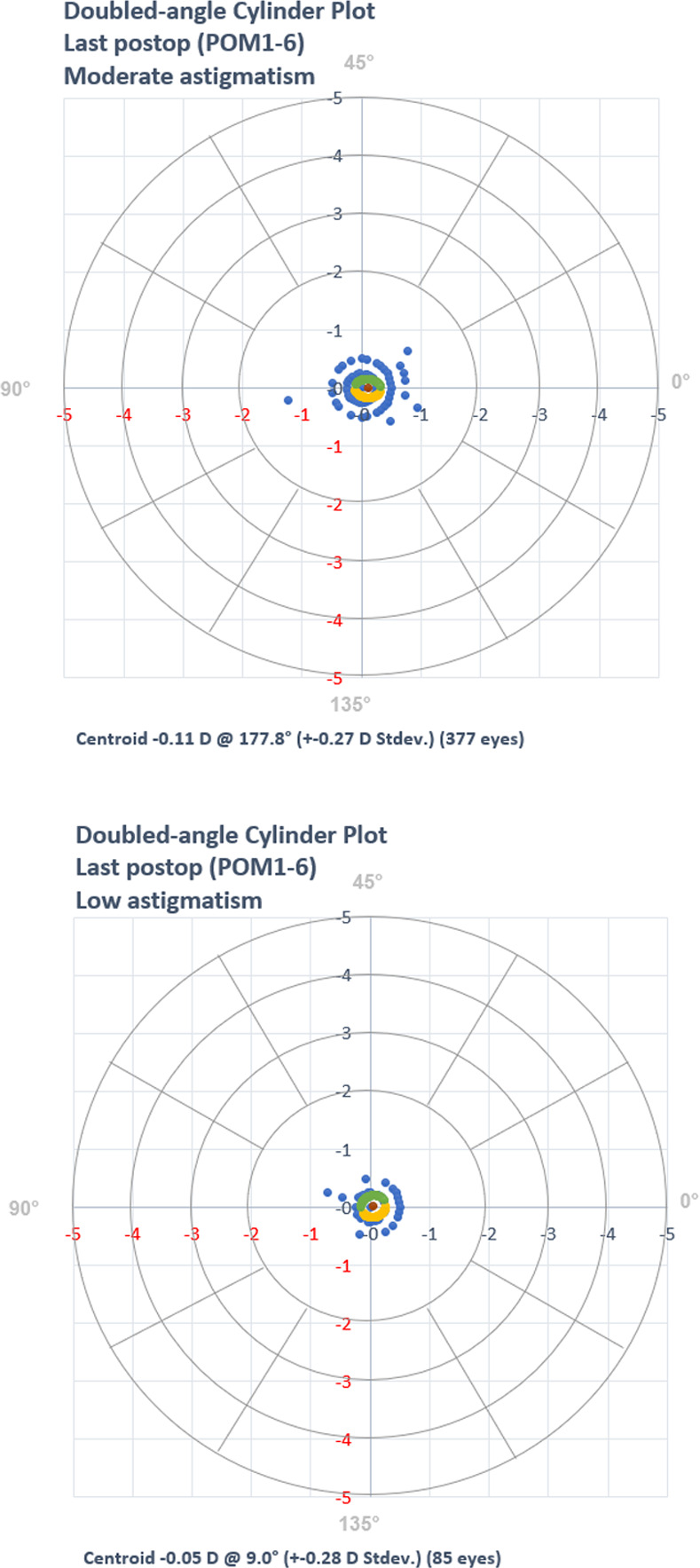
Vector comparison of moderate versus low astigmatism at last visit.

A record review identified 462 eyes of 235 patients who met the inclusion criteria. There were 85 eyes (18.4%) who presented with 0.25 D– 0.5 D of astigmatism (low astigmatism) and 377 eyes (81.6%) that presented with > 0.5 D of astigmatism (moderate astigmatism). No eye needed retreatment because of residual refractive errors during the follow up period. Patient characteristics are summarized in Table 1. The standard refractive surgery graphs are presented in Fig 1. Vector comparison of low astigmatism versus moderate astigmatism is presented in Fig 2.

**Table 1:** Patient demographic and refractive data.

Parameter	Values
Number of eyes (no. of patients)	462 (235)
Percentage male, percentage female	45, 55
Age in years	30.17 ± 4.58 (range: 19 to 39)
Preoperative myopic astigmatism	-1.04 ± 0.77 D (range: 0.00 to -3.00 D)
Preoperative spherical equivalent	-4.96±2.07 D (range: -0.63 to -11.25 D)
Preoperative axis of cylinder	111.82±73.59 (range: 0.00 to 180.00)
No. of eyes with low astigmatism completing follow-up (%)	47 (55%)
No. of eyes lost to follow-up (%)	169 (36.6%)
Final postoperative myopic astigmatism for eyes with complete follow-up (n = 293)	-0.19±0.22 D (range: -1.25 D to 0.50 D)
Final postoperative spherical equivalent for eyes with complete follow-up (n = 293)	-0.10±0.17 D (range: -0.63D to 0.50 D)

D = diopter.

There was no difference in initial refractive cylinder levels or spherical equivalent efficacy between eyes with complete follow-up (n = 293) and those lost to follow-up (n = 169); P>0.05.

**Fig 3:**
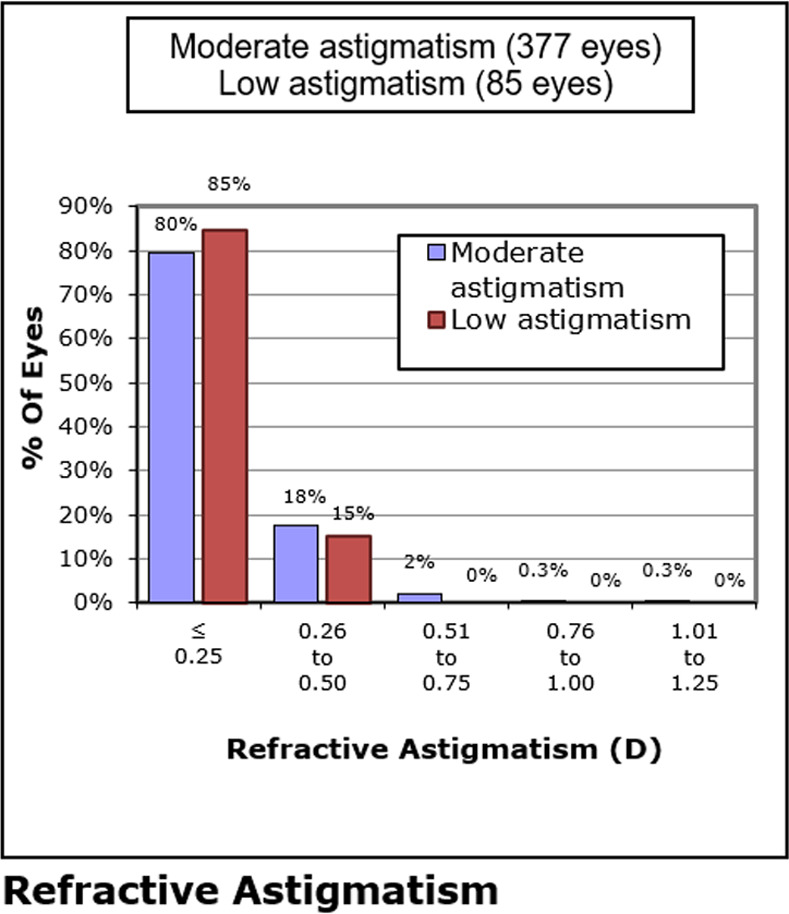
Percentage of both patient groups’ final level of astigmatic refraction at last visit, the difference was not clinically significant. D = diopter.

There were 47 eyes in the low astigmatism group and 246 in the moderate astigmatism group with completed follow-up visit and final visit data. Although eyes with low astigmatism began with significantly lower refractive cylinder levels (P < 0.01), these differences were not present at POM 1 or the final examination (both Ps > 0.05). Further, both groups’ refractive cylinder levels decreased significantly from their respective baselines (both Ps < 0.01). Fig 3 shows the percentage of eyes in each group’s final level of astigmatism.

**Fig 4:**
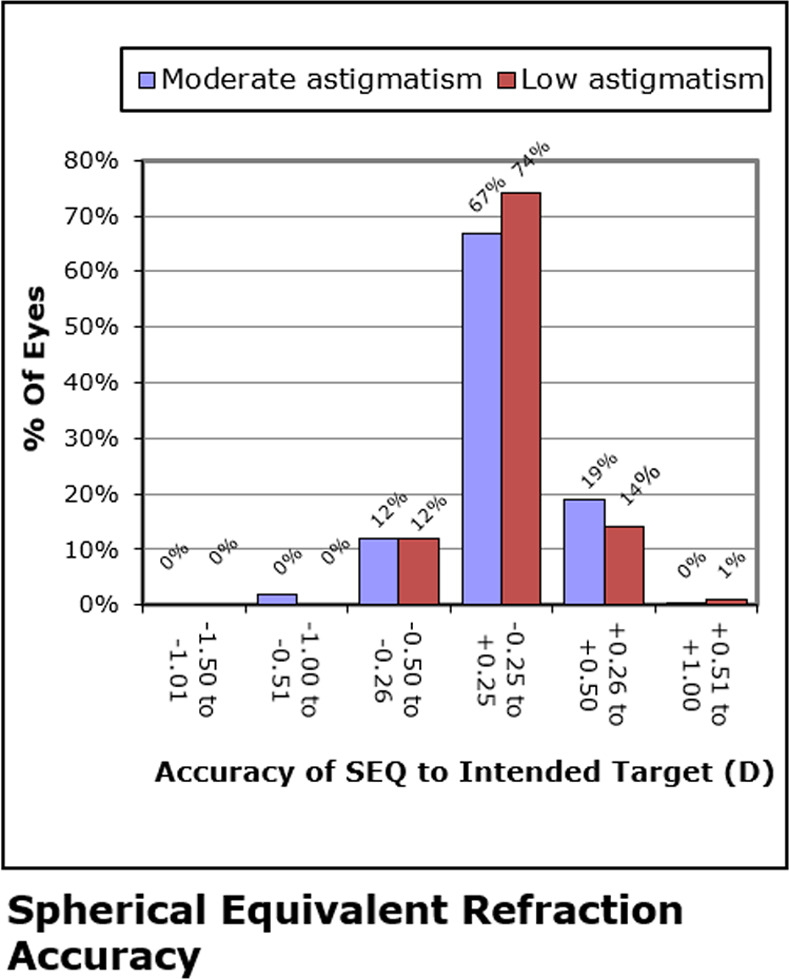
Percentage of both patient groups’ final level of spherical equivalent refraction (SEQ) at last visit, the difference was not clinically significant. D = diopter.

A similar analysis examined the spherical equivalent efficacy for both low and moderate astigmatism groups. The eyes with low astigmatism began with significantly lower spherical equivalent levels (P < 0.05), but these differences were not statistically different at last visit; see Fig 4.

### Efficacy and safety

At POM 1, 425 eyes (92%) had UCVA of 20/20 or better. At final visit, 461 eyes (99.8%) had UCVA of 20/25 or better, 431 eyes (93%) had UCVA of 20/20 or better and 129 eyes (28%) achieved 20/15. When comparing preoperative CDVA to postoperative CDVA at last visit, 8 eyes (1.7%) lost one line and no eyes lost two or more lines. Ninety-seven eyes (21%) gained one line of CDVA. There were no visually significant intraoperative or postoperative complications.

### Predictability

All 462 eyes were within ±1.00D of target at their last visit; 314 eyes (68%) were within ±0.25 D, and 453 eyes (98%) were within ±0.50D. The adjusted logistic regression model showed that attaining UCVA ≥20/20 at the last visit did not differ between the low astigmatism and moderate astigmatism groups (OR, 1.18, 95% CI: 0.605, 2.315). The same test showed no significant difference (P >0.05) in the percentage of low astigmatism eyes with UCVA of 20/20 or better on the last visit (n = 79; 92.9%) than the percentage of moderate astigmatism eyes with UCVA of 20/20 or better on the last visit (n = 352; 93.6%).

### Stability

The average change in refraction from POM 1 to the final visit POM 3–6 of 0.12 D was not statistically significant. Nine eyes (2%) had a change in refraction of more than ±0.50 D.

## Discussion

This study retrospectively reviewed a single center’s refractive outcomes in eyes that underwent SMILE with the most updated energy settings allowed in the United States. Patients in this analysis had a quick recovery time and highly favorable refractive outcomes (98% within ±0.5 D of target). At the last visit, 93% of all eyes achieved UDVA of ≥20/20 with 92% of eyes who followed up at POM 1 already seeing ≥20/20. This study demonstrated no statistical difference between results of patients who presented with low or moderate astigmatism, and it supports SMILE being a safe procedure with no eyes losing over one line of CDVA.

Our results differ from a recent review comparing data submitted to the FDA for the newer SMILE settings to excimer laser technology outcomes that found no significant differences in the two technologies except for a slower visual recovery time [5]. In the FDA study evaluating SMILE, 83.7% of patients had UDVA of 20/20 or better, 0% of patients lost two or more lines of BCVA, 94.0% had a spherical equivalent refraction (SEQ) within ± 0.50D of plano and there was a clinically insignificant change in refraction over the first 6 months postoperatively. Our real-world findings in this study are more closely aligned to findings with excimer laser platforms. While we recognize a head-to-head comparison cannot be made as inclusion criteria and treatment modalities differed in other studies, it is encouraging to note our real-world results are similar to studies on LASIK in terms of visual recovery time and show better efficacy than pivotal SMILE trial results submitted to the FDA.

For example, at POM 1, 65.8% of eyes in the FDA study for SMILE were seeing ≥20/20, 87.5% of eyes in the topography-guided group saw ≥20/20 and 88.0% of eyes saw ≥20/20 in the wavefront-guided group [5]. At POM 1, 92% of our eyes had 20/20 or better vision. This may be due to our increased flexibility to optimize our settings and nomogram than the FDA studies had. It is important to note, however, that the FDA study was restricted to manufacturer recommendations, whereas we were able to adjust our energy as noted in the Methods section and have a site-specific nomogram based on previous results.

Slow visual recovery times with SMILE using older approved energy settings have been reported [14]. Moshirfar et al. [6] reported at 6 months 74% of the patients had BCVA of ≥20/20 and 80% had a SEQ within ± 0.50D of target. More recently, Sia et al. [7] compared SMILE outcomes to LASIK and PRK in active duty service members, albeit also with the older SMILE setting limitations. In that comparison at POM 3, 95% of eyes were seeing 20/20 or better, 94% had a SEQ within ±0.50 D of plano, no eyes lost ≥ 2 lines of BCVA, and 5% had >0.50 D change in SEQ between 1 to 3 months postoperatively. Our results at POM 1 with the low energy settings are similar to the results reported by Sia et al at POM 3, suggesting the lower energy settings may have a positive effect on postoperative recovery time.

Nejad et al. recently published results with the new parameters for SMILE in the US, comparing early results and changes in higher order aberrations (HOA) between previously available US settings (higher total energy) to new US settings (lower total energy) and wavefront-optimized LASIK [10]. They found no significant difference in percentage of eyes with UCVA of 20/20 at POD 1 between low energy SMILE settings (92%) and LASIK (90%). In contrast, at POD 1 the percentage of eyes achieving UCVA of 20/20 from high energy SMILE were significantly worse (37%). At POM 1, our percentage of 20/20 UCVA eyes (92%) was similar to their lower energy SMILE (95%) and LASIK group (95%) but better than their higher energy SMILE (71%). The difference between our results with their lower energy SMILE results may be secondary to our higher average and larger range of attempted SEQ correction which may have led to more variable healing times. Additionally, we included eyes that had preoperative BCVA of 20/25, whereas eyes with BCVA worse than 20/20 were excluded from their study.

Our findings also differ in a few other key points. We had a larger dataset, specifically addressed eyes with low astigmatism and did not analyze HOAs. Nejad et al. treated eyes with 0.5D of astigmatism with LASIK if full cylinder correction was subjectively preferred over SEQ [10]. Patients in their study were treated with SEQ if they had no preference and topography was consistent with cylinder of 0.5 D or less. There was no further analysis of the outcomes of this methodology noted. In our study, we treated eyes with low astigmatism of 0.25 D or 0.50 D with SMILE and found no statistically or clinically significant differences in outcomes between those eyes and in eyes with moderate astigmatism. Despite these differences, our similar POM 1 results support their conclusion that recovery time with lower energy SMILE now available in the US is significantly faster than previously available higher energy SMILE.

We encourage future studies to analyze outcomes in patients with low astigmatism to independently confirm our results and our methodology for treating these eyes. Future studies can aspire to prospectively evaluate the outcomes in patients with low astigmatism in comparison to a LASIK control group, compare results between treatment determined by patient’s subjective preference versus topography, as well as set a stricter and longer follow-up period to assert stability.

Non-published data from South Korea on more than 63,000 eyes suggests the need for enhancement post-SMILE with nomogram adjustment is about one-third the rate of needed enhancements in post-LASIK eyes. [Charters, L. SMILE procedure offers low enhancement rate after nomogram adjustment. Ophthalmology Times. Mar 15, 2021, Volume 46, Issue 5.] Our results support that, as none of our patients needed retreatment. The possible reduced enhancement rate with SMILE may be attributed to the theoretically increased stability and predictability attributed to a closed system treatment and less biomechanical disruption. Nevertheless, we suggest to cautiously interpret our specific enhancement rate report due to the short follow-up period. Previous peer-reviewed studies show mean time for enhancement after SMILE to be around 10 months [15, 16]. These studies suggest similar enhancement rates between SMILE and LASIK but do not specify nomogram adjustments.

There are several additional limitations in our study, including those inherent to a retrospective review. For example, we do not have data points for many of the eyes and there is no control group. We have no explanation about those who were lost to follow-up compared to those who attended every postoperative visit, except to theorize that people who were satisfied with their postoperative vision may not have felt a need to continue postoperative care. As previously stated, our overall follow-up time (one to six months) was relatively short, which may have affected our results. Several studies, however, have shown excellent stability starting at POM 1 with SMILE which can likely be extrapolated to our patient population [17, 18]. Importantly, the coronavirus pandemic and limitations put on postoperative clinic visits for elective surgery in the State of California prevented some patients from being able to complete follow-up visits. It is noted that the initial spherical equivalent refraction was statistically lower in our low astigmatism group, and that may have influenced the comparability of final results between our low astigmatism and moderate astigmatism groups.

These potential limitations do not negate our findings that SMILE with U.S.-approved low energy settings has a short visual recovery time with both excellent visual outcomes and safety data for patients with moderate and low astigmatism.

## Supporting information

S1 Dataset (XLS)
